# In Situ Functionalization of Iron Oxide Particles with Alginate: A Promising Biosorbent for Retention of Metal Ions

**DOI:** 10.3390/polym13203554

**Published:** 2021-10-15

**Authors:** Alina-Roxana Lucaci, Dumitru Bulgariu, Laura Bulgariu

**Affiliations:** 1 Department of Environmental Engineering and Management, “Cristofor Simionescu” Faculty of Chemical Engineering and Environmental Protection, Technical University Gheorghe Asachi of Iasi, 700050 Iasi, Romania; alina-roxana.lucaci@tuiasi.ro; 2 Department of Geology and Geochemistry, Faculty of Geography and Geology, “Al. I. Cuza” University of Iasi, 700506 Iasi, Romania; dbulgariu@yahoo.com; 3 Collective of Geography, Filial of Iasi, Romanian Academy, 700506 Iasi, Romania

**Keywords:** biosorption, alginate, functionalized iron oxide particles, metal ions retention

## Abstract

In this study, alginate extracted from marine algae biomass was used for the functionalization of iron oxide particles obtained in situ. This procedure ensured a complete recovery of the alginate from the aqueous solution obtained after extraction and allowed the preparation of a new biosorbent. The obtained iron oxide microparticles functionalized with alginate (Alg-Fe_3_O_4_-MPs) were analyzed (FTIR spectrometry, energy dispersive X-ray spectroscopy and scanning electron microscopy), and their biosorptive performance was tested for the removal of Cu(II), Co(II) and Zn(II) ions. The optimal conditions were established as pH = 5.4, adsorbent dosage of 2 g/L, contact time of minimum 60 min and room temperature (23 ± 1 °C). The retention of metal ions was quantitative (99% for Cu(II), 89% for Co(II) and 95% for Zn(II)) when the concentration of metal ions was less than 0.80 mmol M(II)/L. The Langmuir model was found to be the best fitted model for the equilibrium data, while biosorption kinetics followed the pseudo-second order model. Biosorption processes were spontaneous (ΔG^0^ < 0), endothermic (ΔH^0^ > 0), and accompanied by an increase in entropy (ΔS^0^ > 0). The high maximum biosorption capacity of Alg-Fe_3_O_4_-MPs and its good regeneration highlight the potential of this biosorbent for applications in decontamination processes.

## 1. Introduction

Efficient treatment of industrial effluents before their discharge into the environment is one of the main ways to reduce the negative impact of industrial activities on the quality of ecosystems. This is because industrial effluents contain many organic compounds and/or metal ions in a wide range of concentrations, which, once in the environment, contribute significantly to environmental pollution [1]. It is well known that organic compounds such as pharmaceuticals and dyes affect the flora and fauna of surface waters and are mainly responsible for their eutrophication [2]. On the other hand, metal ions are not biodegradable, persist over time and can accumulate along the food chain [3]. Consequently, the removal of organic and inorganic pollutants from industrial effluents prior to their discharge into the environment has become an essential condition for the conduct of industrial activities, in accordance with the principles of clean production [1].

A large number of studies in the literature [4,5,6,7] have reported technological solutions (including chemical precipitation, membrane processes, catalytic oxidation, ion exchange, and more) to reduce the content of metal ions and organic compounds in industrial effluents. Unfortunately, many of these methods have some important limitations (moderate efficiency and selectivity, high operating costs, etc.), which has led to a search for alternatives in order to minimize them [8]. In contrast, biosorption can be considered a more appropriate method for removing organic and inorganic pollutants from industrial effluents, due to its cost effectiveness, adaptability to different experimental conditions and different types of contaminants, excellent recyclability, and waste minimization [9,10,11]. In addition, many materials used as biosorbents are easy to purchase, inexpensive and require only a few simple operations before they can be used in decontamination processes.

Particular attention has been paid to the use of marine algae biomass for these type of applications [12,13,14]. Their high availability in many regions of the world, cultivation without the need for agricultural land use, simple and inexpensive procedures for their preparation as biosorbents, and the wide variety of functional groups on their surface are just a few of the advantages that have made marine algae biomass frequently used in biosorption processes [15]. Unfortunately, with a few exceptions, most marine algae biomass has only a moderate biosorption capacity, especially for metal ions, and therefore a single biosorption step is insufficient for the quantitative removal of these pollutants from aqueous media [16]. This drawback, together with low mechanical strength and small particle size, are the main reasons why marine algae biomass is still quite sparsely used in industrial wastewater treatment processes.

In a previous study [17], we showed that alginate extracted from marine algae biomass can be a much more effective biosorbent in removing metal ions from aqueous solution. This is due to the fact that alginate, which is an organic biopolymer, has numerous available functional groups [18] which can bind metal ions from aqueous media. Moreover, because it is a natural polymer, the alginate extracted from marine algae biomass can be considered an environmentally friendly material. 

The extraction of alginate from marine algae biomass can be done quite easily in alkaline media; the extraction procedure was detailed by Wang et al., 2018 [19]. This procedure briefly involves two stages: (i) treatment of algae biomass with a concentrated alkaline solution (most often a 1N NaOH solution), which has the role of solubilizing the alginate macromolecules from algae biomass; and (ii) precipitation of dissolved alginate macromolecules by adding electrolytes (1M CaCl_2_ solution), followed by filtration of the solid phase. If the efficiency of the first stage of the extraction procedure can be rigorously controlled by appropriate selection of experimental conditions (type and concentration of alkaline solution, contact time, temperature, etc.), additional precautions are still required in carrying out the second stage due to the high solubility of alginate in water [20]. To minimize undesirable consequences, the addition of water-miscible organic solvents such as methanol, ethanol, or isopropyl alcohol is proposed in the literature [21,22] in order to reduce the solubility of alginate. Although this alternative can significantly improve the efficiency of the alginate extraction process, the additional use of organic solvents increases costs and has negative effects on the environment. 

The solution proposed by us in this study is the use of the extract containing alginate (obtained after the first stage of extraction) for the functionalization of iron oxide particles prepared in situ. In this way the loss of alginate due to its solubility is minimal, most of these macromolecules being immobilized on the surface of iron oxide particles and not requiring the use of organic solvents. Thus, the obtained material should have high biosorptive performances due to: (i) its micro-particle characteristics, conferred by iron oxide particles; and (ii) the large number of superficial functional groups, due to the presence of alginate. 

Therefore, in this study, alginate extracted from marine algae biomass in alkaline media was used for the functionalization of iron oxide particles obtained in situ. This procedure allowed the preparation of a new biosorbent material that can be used in environmental decontamination processes. The obtained biosorbent, Alg-Fe_3_O_4_-MPs, was detailed, characterized and its biosorptive performances were tested for the removal of Cu(II), Co(II) and Zn(II) ions from aqueous media, in batch systems. Based on the experimental data, the isotherm, kinetic and thermodynamic characteristics of the biosorption processes were analyzed. In addition, desorption studies were performed to highlight the potential reusage of this material.

## 2. Materials and Methods

### 2.1. Chemicals

Chemical reagents FeCl_2_, FeCl_3_, HNO_3_, NaOH, were of analytical grade and were purchased from Chemical Company (Romania). The metal sulphate salts (CuSO_4_, CoSO_4_ and ZnSO_4_) were purchased from Sigma Aldrich, and were used as received for the preparation of stock solutions of metal ions (10^−2^ mol/L) by dissolving the solid salts in distilled water. All working solutions of each metal ion were obtained by fresh dilution from the stock solution.

### 2.2. Preparation and Characterization of Biosorbent (Alg-Fe_3_O_4_-MPs)

The extraction of alginate from marine algae biomass as well as its identification and characterization were detailed in a previous study [23]. The preparation of the Alg-Fe_3_O_4_-MPs biosorbent involves two steps: (1) solutions of FeCl_2_ (3.976 g) and FeCl_3_ (10.812 g) were obtained separately by dissolving FeCl_2_ and FeCl_3_ salts in 100 mL of alginate solution, obtained after extraction; (2) these two solutions were then mixed at a volume ratio of 2:1, and the pH of the mixture was adjusted at 11 by adding a 1 N NaOH solution and stirred vigorously for 1 h. The obtained solid micro-particles were then filtrated in vacuum, washed several times with distilled water, and dried in air. The characterization of the Alg-Fe_3_O_4_-MPs biosorbent was done by FTIR spectrometry (FTIR Bio-Rad Spectrometer, 400–4000 cm^−1^; resolution of 4 cm^−1^, KBr pellet technique) in order to highlight the presence of superficial functional groups, and by scanning electron microscopy combined with energy dispersive X-ray analyzed (SEM/EDAX Hitach S3000N, 20 kV) in order to examine the surface morphology. In addition, the surface area of the Alg-Fe_3_O_4_-MPs biosorbent was determined by N_2_ adsorption/desorption analysis; the obtained value was 136.18 m^2^/g.

### 2.3. Biosorption Experiments

The biosorption experiments were performed in batch systems by mixing 25 mL of metal ions solution (Cu(II), Co(II) and Zn(II)) under well-defined experimental conditions. The variation intervals of experimental parameters were as follows: initial solution pH = 2.4–6.2; biosorbent dosage = 2.0–20.0 g/L, initial concentration of metal ions = 0.2–2.8 mmol M(II)/L, contact time = 5–180 min, and temperature = 7, 23 and 40 °C. The values of the experimental parameters corresponding to the maximum biosorption efficiency were considered optimal. After completion of the biosorption procedure, the two phases were filtrated and the metal ions concentration in the aqueous solution was analyzed by Atomic Absorption Spectrometry (AAS NovAA 400P Spectrometer, acetylene/air flame). The biosorption parameters, namely biosorption capacity (q, mmol/g) and removal percent (R, %), were calculated using the equations

(1) $$q=\frac{(c_{0}-c)\cdot V}{m}$$

(2) $$R=\frac{c_{0}-c}{c_{0}}\cdot 100$$

where *c_o_* and *c* are the initial and equilibrium metal ions concentration in solution (mmol/L), *V* is volume of solution (L), and *m* is the mass of Alg-Fe_3_O_4_-NPs used in the experiment (g).

### 2.4. Equilibrium and Kinetic Models

Kinetic data on Cu(II), Co(II) and Zn(II) biosorption by Alg-Fe_3_O_4_-MPs were analyzed using the equations of the pseudo-first order model (Equation (3)), pseudo-second order model (Equation (4)) and intra-particle diffusion model (Equation (5)) [24,25,26]

(3) $$\log(q_{e}-q_{t})=\log q_{e}-k_{1}\cdot t$$

(4) $$\frac{t}{q_{t}}=\frac{1}{k_{2}\cdot q_{e}^{2}}+\frac{t}{q_{e}}$$

(5) $$q_{t}=k_{diff}\cdot t^{1/2}+c$$

where *q_e_*, *q_t_*—biosorption capacity at equilibrium and at time *t,* (mmol/g); *k_1_*—rate constant of pseudo-first order kinetics model, (1/min); *k_2_*—pseudo-second order rate constant, (g/mmol min); *k_diff_*—intra-particle diffusion rate constant, (mmol/g min^1/2^), *c*—concentration of metal ions from solution at equilibrium, (mmol/L).

The equilibrium data obtained on Cu(II), Co(II) and Zn(II) biosorption by Alg-Fe_3_O_4_-MPs were examined using Langmuir (Equation (6)), Freundlich (Equation (7)), and Temkin (Equation (8)) isotherm models. The mathematical equations of these models [27,28] are

(6) $$\frac{1}{q}=\frac{1}{q_{\max}\cdot K_{L}}\cdot \frac{1}{c}$$

(7) $$\log q=\log K_{F}+\frac{1}{n}\cdot \log c$$

(8) $$q=B\ln A_{T}+B\ln c$$

where *q*—biosorption capacity at equilibrium, (mmol/g); *q_max_*—maximum biosorption capacity, (mmol/g); *K_L_*—Langmuir constant, (L/g); *c*—equilibrium concentration of metal ions in solution, (mmol/L); *K_F_*—Freundlich constant, (L/g); *n*—heterogeneity factor; *A_T_*—Temkin isotherm equilibrium binding constant, (L/g); *B*—constant correlated with the heat of biosorption process, (J/mol).

### 2.5. Desorption Experiments

Three samples of 0.5 g of Alg-Fe_3_O_4_-MPs biosorbent, each loaded with known amounts of metal ions of Cu(II), Co(II) or Zn(II), were used for the desorption experiments. From each sample, 0.1 g was treated with 5 mL of 0.1 N HNO_3_ solution, stirred for 3 h, and filtered. The metal ions concentration, analyzed as presented above, was then used for the calculation of desorption percent (Desorption %) according to the equation

(9) $$\%\;\mathrm{Desorption}=\frac{c_{d}}{q\cdot m}\cdot 100$$

where *c_d_* is the concentration of metal ions desorbed from Alg-Fe_3_O_4_-MPs (mmol/L), *q* is the biosorption capacity (mmol/g), and *m* is the amount of Alg-Fe_3_O_4_-MPs used for desorption (g).

The solid biosorbent samples obtained after desorption were treated with 0.1 N NaOH solution to a neutral pH, washed, dried and used in the next biosorption cycle. Five biosorption/desorption cycles were performed for each metal ion, and desorption percent was calculated in each case according to Equation (9). 

The biosorption and desorption experiments were carried out in triplicate, and the mean values of the experimental data were used in graphs and calculations. Data were analyzed by ANOVA with unpaired *t*-test. *p*-values of less than 0.05 were considered significant.

## 3. Results and Discussion

### 3.1. Characterization of Alg-Fe_3_O_4_-MPs Biosorbent

The preparation methodology of Alg-Fe_3_O_4_-MPs biosorbent was designed so as to solve two problems, namely: (i) to allow the quantitative removal of the extracted alginate from aqueous solution, and (ii) to allow obtaining a material with biosorptive properties that is stable over time. These are the main reasons why it was preferred that the iron salts be dissolved separately and directly in the alginate solution, and that the functionalized microparticles be obtained in situ (see Section 2.2). The quantitative removal of the extracted alginate from aqueous solution was evaluated by the oxidability index (CCO). CCO index is an indicator that allows the evaluation of the content of organic compounds in an aqueous solution via the oxygen demand necessary for their oxidation [29]. If the value of this index in the solution obtained immediately after alginate extraction was 446.86 mg O_2_/L, after filtration of Alg-Fe_3_O_4_-MPs, the value would be significantly lower (33.51 mg O_2_/L). The decrease of the CCO index value by more than 92% shows that the formation of Alg-Fe_3_O_4_-MPs determines the binding of the alginate macromolecules from the aqueous solution. In addition, the obtained solid material has considerable stability over time, because after 30 days of contact with distilled water, the value of the CCO index did not change by more than 3.5% (data not shown). Therefore, this procedure allows the process of alginate extraction from algae biomass to be carried out in a simple and environmentally friendly manner.

**Figure 1:**
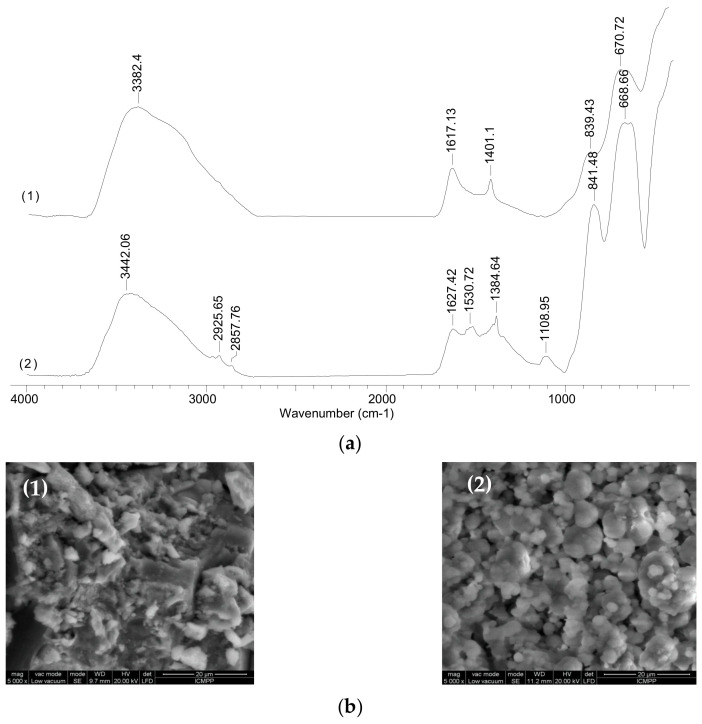

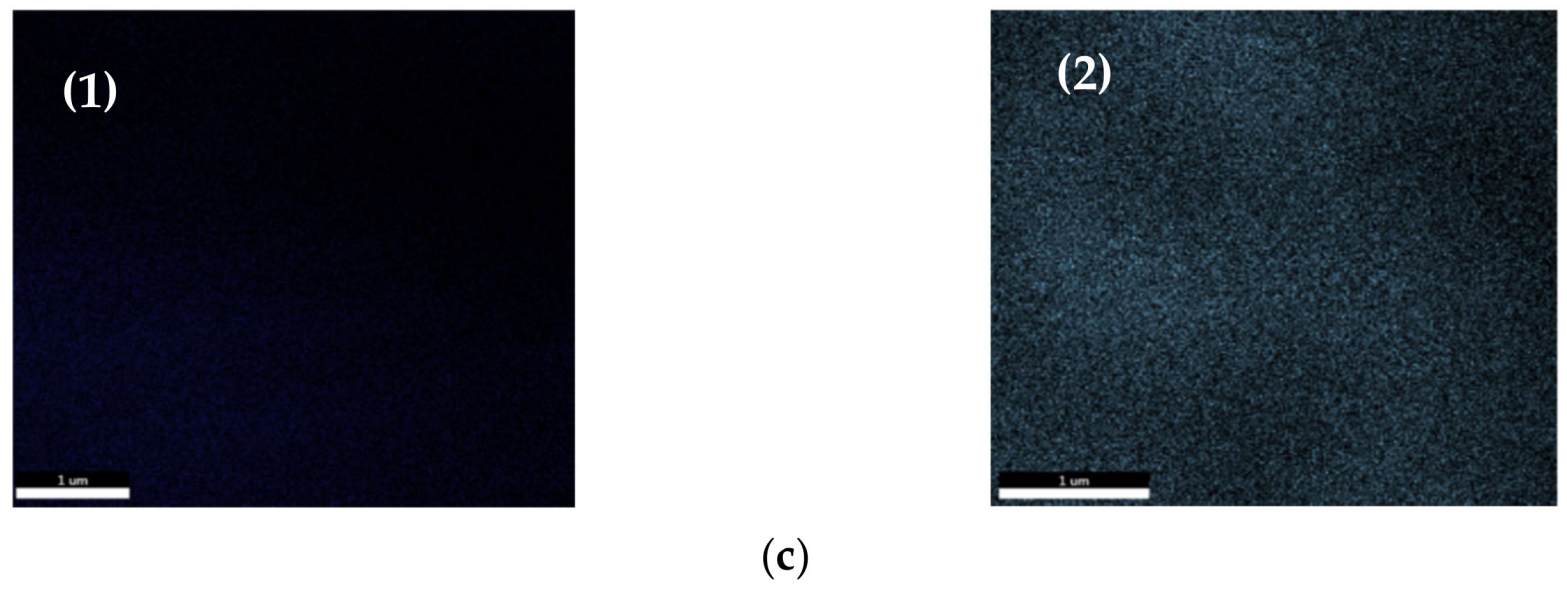
(**a**) FTIR spectra, (**b**) SEM images and (**c**) mapping images of Fe_3_O_4_-MPs (1) and Alg-Fe_3_O_4_-MPs (2).

To highlight the structural changes, FTIR spectra (Figure 1a) were recorded for the solid materials obtained by mixing iron salts solutions prepared in both distilled water (Fe_3_O_4_-MPs—spectra (1)) and in alginate solution (Alg-Fe_3_O_4_-MPs—spectra (2)) into alkaline media (pH = 11). 

It can be easily seen from Figure 1a that in spectrum (2) there are several additional absorption bands compared to spectrum (1). Thus, the bands from 2925–2857 cm^−1^, which correspond to the C–H bonds from hydrocarbon radicals, and the absorption bands from 1530, 1384 and 1108 cm^−1^, which can be attributed to the stretching vibrations of the C–O bonds from the oxygenated compounds (carbonyl and carboxyl), indicate that the alginate has been successfully bound on the surface of Fe_3_O_4_-MPs. Moreover, the absorption band from 3382 cm^−1^ in spectrum (1) (stretching vibrations of the H–O bond) moves at a higher wave number (3442 cm^−1^—in spectrum (2)). The displacement of this absorption band after alginate binding is probably determined by two factors: (i) the exclusion of water molecules from the Fe_3_O_4_-MPs surface as a consequence of the binding of alginate macromolecules, and (ii) the presence of hydroxyl groups from the alginate structure. Based on these observations, it can be seen that the obtained material has numerous and varied functional groups on its surface and that Alg-Fe_3_O_4_-MPs has potential to become an effective biosorbent for the removal of metal ions from aqueous media. 

The morphology of the surface of the Alg-Fe_3_O_4_-MPs adsorbent and the distribution of carbon atoms on it were examined using the SEM/EDAX technique; the obtained images are illustrated in Figure 1b,c. As can be seen from Figure 1b, Alg-Fe_3_O_4_-MPs biosorbent is composed of sphere-like particles, with a non-uniform surface and relatively large pore volume and size. The non-uniform surface of the Alg-Fe_3_O_4_-MPs biosorbent and the relatively large range of particle sizes is probably determined by the alginate deposition, formed in situ, which covers the surface of the Fe_3_O_4_-MPs. Thus, an “outer coating” of Fe_3_O_4_-MPs is obtained which has functional groups. The formation of the outer alginate layer was highlighted by the EDAX images (Figure 1c), where the relative uniform distribution of carbon atoms on the Alg-Fe_3_O_4_-MPs surface compared to the Fe_3_O_4_-MPs surface can be observed. In addition, due to this outer layer of alginate, Alg-Fe_3_O_4_-MPs particles tend to form much larger aggregates (Figure 1b), which can be easily separated by vacuum filtration.

**Figure 2:**
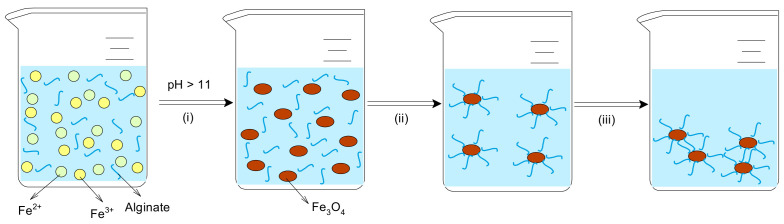
Schematic illustration of the formation of Alg-Fe_3_O_4_-MPs.

Taking into account all of these experimental observations, the formation of Alg-Fe_3_O_4_-MPs can be represented by a succession of three elementary stages (Figure 2), namely: (i) precipitation of Fe_3_O_4_-MPs which will represent the core of the new adsorbent—this elementary process takes place spontaneously under the mentioned experimental conditions; (ii) binding of alginate macromolecules to the surface of formed Fe_3_O_4_-MPs—this process is favoured by the exclusion of water molecules from the Fe_3_O_4_-MPs surface during formation; (iii) aggregation of the obtained Alg-Fe_3_O_4_-MPs—this process is probably due to the hydrogen bonds that may occur between functional groups of alginate on the Alg-Fe_3_O_4_-MPs surface. 

In this way, a solid material is obtained which is easily separated from aqueous media (by vacuum filtration) and has a large number of functional groups (predominantly OH) on its surface. These characteristics are very important for biosorbent in biosorption processes, and therefore Alg-Fe_3_O_4_-MPs has been tested for the removal of metal ions from aqueous solutions. 

### 3.2. Biosorptive Performances of Alg-Fe_3_O_4_-MPs

In this study, the performance of Alg-Fe_3_O_4_-MPs biosorbent has been examined for the removal of Cu(II), Co(II) and Zn(II) ions from aqueous media. The choice of these metal ions for experimental studies was made taking into account their frequent use in industrial processes and their chemical properties.

#### 3.2.1. Selection of Optimal Conditions

**Figure 3:**
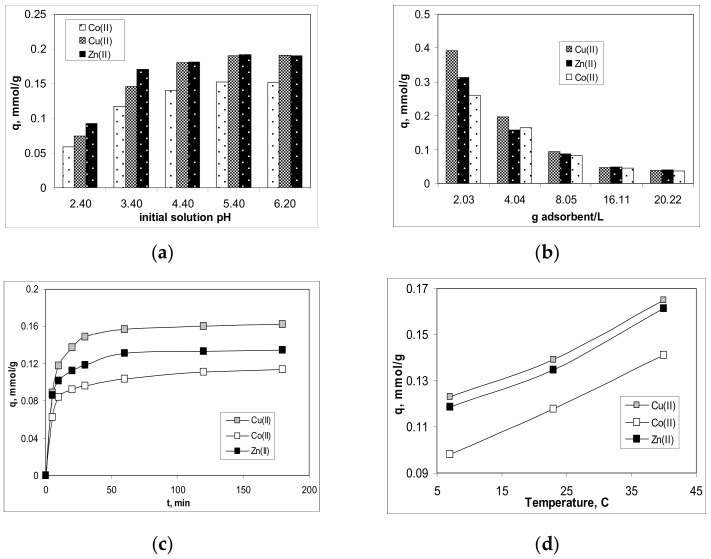
Effect of initial solution pH (**a**), adsorbent dosage (**b**), contact time (**c**) and temperature (**d**) on biosorption of Cu(II), Co(II) and Zn(II) ions on Alg-Fe_3_O_4_-MPs.

The selection of the optimal experimental conditions is an important step in establishing the performance of a given biosorption system [30]. In this study, four experimental parameters were considered: initial solution pH, adsorbent dosage, contact time and temperature. The initial solution pH and adsorbent dosage are two very useful parameters in describing the interactions that take place during the adsorption process, while the contact time and temperature are mainly related to adsorption costs [31]. The experimental results obtained in the study of the influence of each experimental parameter are presented in Figure 3.

The variation of the biosorption capacity of Alg-Fe_3_O_4_-MPs for Cu(II), Co(II) and Zn(II) ions as a function of initial solution pH is shown in Figure 3a. For all studied metal ions, the biosorption capacity of Alg-Fe_3_O_4_-MPs increased with increasing initial solution pH, and reached a maximum value in the pH range between 5.40 and 6.20. This variation suggests the existence of predominantly electrostatic interactions between metal ions and superficial functional groups during the biosorption processes. At the lowest initial solution pH (pH = 2.4), the high concentration of protons competes with the metal ions for the binding sites on the adsorbent surface. Thus, the obtained biosorption capacities are low (0.07 mmol/g for Cu(II), 0.06 mmol/g for Co(II) and 0.07 mmol/g for Zn(II)), and the removal percentages are not higher than 35% for all studied metal ions. The increase of the initial solution pH (pH > 2.4) determined the increase of the biosorption capacity of Alg-Fe_3_O_4_-MPs (see Figure 3a). This increase is evident in the pH range of 2.4–3.4, after which the values of this parameter remain almost constant, and the removal percentages are higher than 87% for all studied metal ions. Such a variation clearly shows that if the speciation of metal ions does not change, the biosorption capacity of Alg-Fe_3_O_4_-MPs is very little influenced by the variation of initial solution pH. This observation is important from two points of view. First, it shows that on the surface of Alg-Fe_3_O_4_-MPs most of functional groups are dissociated (probably due to the preparation procedure) and thus the variation of initial solution pH does not influence their availability to interact with metal ions from the aqueous solution. Secondly, from a practical point of view, when using such biosorbents on an industrial scale it is useful for rigorous control of the pH of effluents prior to treatment to not be necessary. Considering these results (Figure 3a), the optimal initial solution pH was set at 5.4, and was used in the following experiments. 

The effect of different amounts of Alg-Fe_3_O_4_-MPs on the biosorption efficiency of Cu(II), Co(II) and Zn(II) ions was also examined, and the obtained values of biosorption capacities are presented in Figure 3b. In the range of 2.0–20.0 g/L, the biosorption capacity of Alg-Fe_3_O_4_-MPs decreased from 0.393 to 0.038 mmol/g for Cu(II), 0.261 to 0.036 mmol/g for Co(II), and 0.312 to 0.041 mmol/g for Zn(II), respectively. In addition, in the same range of biosorbent dosage, the removal percentages vary much less (from 96.15 to 99.83% for Cu(II), 93.16 to 97.08% for Co(II), and 95.87 to 99.45% for Zn(II), respectively). These variations show that although most Alg-Fe_3_O_4_-MPs binding sites are located on the surface of the biosorbent and are available to interact with metal ions, increasing the amount of biosorbent does not significantly improve biosorption efficiency, most likely due to particle agglomeration (also highlighted by SEM images). Under these conditions, a biosorbent dose of 2.0 g/L was considered optimal, and was used in all other experiments.

Figure 3c shows the influence of contact time on the adsorption of Cu(II), Co(II) and Zn(II) ions on Alg-Fe_3_O_4_-MPs biosorbent. Under the mentioned experimental conditions, the biosorption capacity of Alg-Fe_3_O_4_-MPs increases with increasing contact time over the entire time interval. For all studied metal ions, the biosorption efficiency increases significantly up to 60 min of contact time, and then this increase is much slower, suggesting that the biosorption processes have reached equilibrium and a constant rate. Such a variation is consistent with the general description of the biosorption processes and is mainly due to the large number of active sites on the biosorbent surface in the initial stage of contact between the two phases [30,32]. As the contact time increases, the number of free biosorption sites decreases, the binding of metal ions from the aqueous solution is more difficult and the rate of the biosorption processes consequently decreases. This is a typical behaviour for biosorption systems in which the binding of metal ions from aqueous solution is done predominantly by electrostatic interactions [33], and is favoured by the large surface area of Alg-Fe_3_O_4_-MPs. Therefore, an optimal contact time of 120 min. was selected for subsequent experiments; this value of contact time ensures the quantitative removal of metal ions (90.73% for Cu(II), 82.82% for Co(II) and 87.45% for Zn(II), respectively). In addition, the achievement of maximum biosorption capacity and a constant rate in a relatively short time highlights the potential advantages of Alg-Fe_3_O_4_-MPs for large-scale applications.

Figure 3d illustrates the variation of the biosorption capacities of Alg-Fe_3_O_4_-MPs for Cu(II), Co(II) and Zn(II) ions at three different temperatures (7, 23 and 40 °C), while the other experimental conditions were kept constant at the optimal values previously established. When the temperature increases from 7 to 40 °C, the biosorption capacity of Alg-Fe_3_O_4_-MPs increased for all studied metal ions (from 0.12 to 0.17 mmol/g for Cu(II), 0.09 to 0.14 mmol/g for Co(II), 0.12 to 0.16 mmol/g for Zn(II), respectively), indicating that higher temperatures favour the biosorption of metal ions, and that these processes are endothermic. This variation is mainly determined by two factors: (i) the increase of the thermal agitation of the metal ions in aqueous solution, and (ii) the “expansion” of the pore size on the biosorbent surface, which determines the increase of biosorption efficiency [32,33]. However, compared to the biosorption capacities obtained at ambient temperature (23 °C), the decrease or increase of the temperature (7 and 40 °C) determines a variation in them of only 18% for Cu(II), 16% for Co(II) and 17% for Zn(II). Therefore, the ambient temperature was selected as optimal for the biosorption of Cu(II), Co(II) and Zn(II) ions on Alg-Fe_3_O_4_-MPs, because it is economically advantageous.

#### 3.2.2. Isotherm Modelling 

**Figure 4:**
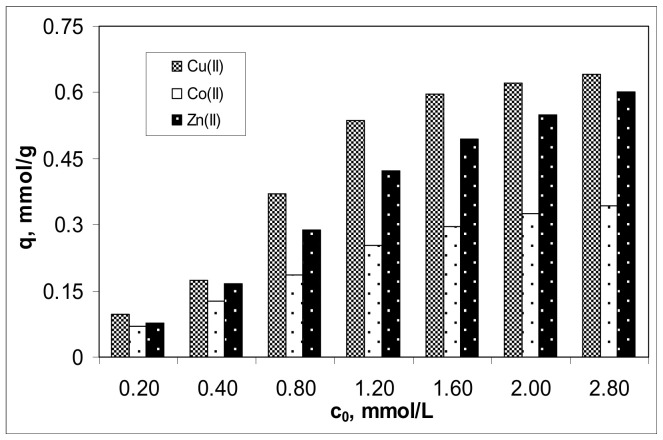
Effect of initial metal ions concentration on biosorption of Cu(II), Co(II) and Zn(II) ions on Alg-Fe_3_O_4_-MPs. (Experimental conditions: pH = 5.4; adsorbent dosage = 2.0 g/L; contact time = 24 h; temperature = 23 °C).

The influence of initial concentration of Cu(II), Co(II) and Zn(II) on the biosorption on Alg-Fe_3_O_4_-MPs was examined in a concentration range of metal ions between 0.2 and 2.8 mmol/L, under optimal experimental conditions. The obtained values of the biosorption capacities are illustrated in Figure 4.

Increasing the initial concentration of metal ions from 0.2 to 2.8 mmol/L led to an increase in the biosorption capacity of Alg-Fe_3_O_4_-MPs from 0.09 to 0.64 mmol/g for Cu(II), from 0.07 to 0.34 mmol/g for Co(II), and from 0.08 to 0.60 mmol/g for Zn(II), respectively. In the studied concentration range, the efficiency of the biosorption process increased in the order: Cu(II) > Zn(II) > Co(II), which is similar to the order of hydrolysis constant values (pK_h_: 7.53 for Cu(II), 9.60 for Zn(II) and 11.35 for Co(II)) [34]. This means that Cu(II) ions will interact most efficiently with the functional groups of Alg-Fe_3_O_4_-MPs, followed by Zn(II) ions and then Co(II) ions, as is easily observed in Figure 4. 

On the other hand, it can be seen from Figure 4 that the dependences q vs. c_0_ are nonlinear for all studied metal ions. In the range of low metal ions concentrations (0.2–1.2 mmol/L), the values of biosorption capacities increase significantly (more than five times for Cu(II) and Zn(II), and 3.5 times for Co(II)), while in the range of high metal ions concentrations (1.2–2.8 mmol/L), the increase of the biosorption capacities is less pronounced (only 19% for Cu(II), 22% for Zn(II) and 32% for Co(II)). Such nonlinear variation is mainly determined by the ratio between the number of functional groups of the biosorbent (which is constant) and the number of metal ions in the aqueous solution (which increases with increasing initial concentration) [6], and has two important consequences. The first consequence is theoretical, and shows that the biosorption processes involve predominantly chemical interactions between metal ions and the functional groups of the biosorbent. Thus, if the ratio between the number of functional groups and the number of metal ions in the solution is high (low concentration range), the metal ions interact easily and the biosorption capacity increases. When this ratio decreases (high concentration range), the number of functional groups becomes insufficient to bind all metal ions from the aqueous solution. The surface of the biosorbent becomes saturated, and the values of the biosorption capacities increase more slowly. The second consequence is of a practical nature, and shows that the efficient removal of the studied metal ions is achieved only at low concentrations (up to 0.8 mmol/L, in this case), when the values of removal percent are higher than 90% (99% for Cu(II), 89% for Co(II) and 95% for Zn(II)). At higher values of the initial concentration of metal ions, their efficient removal requires two or more biosorption steps. This limitation (initial metal ions concentration less than 0.8 mmol M(II)/L) is acceptable for real industrial effluents and highlights the potential applicability of this biosorbent in the treatment of wastewater.

**Figure 5:**
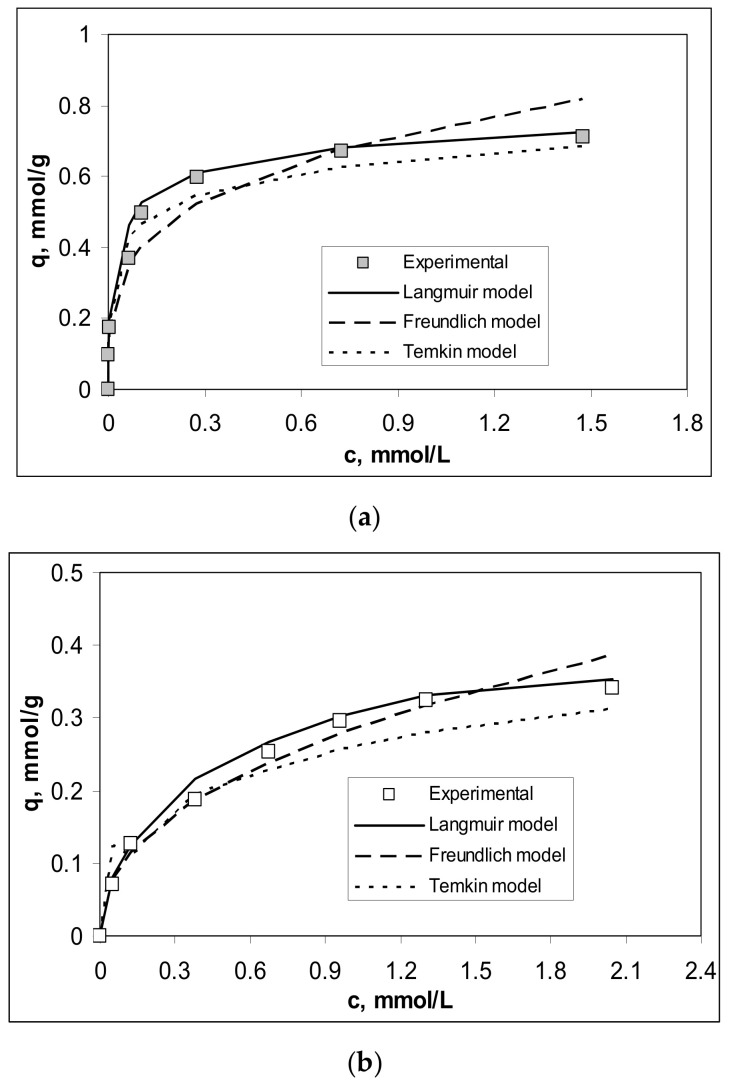

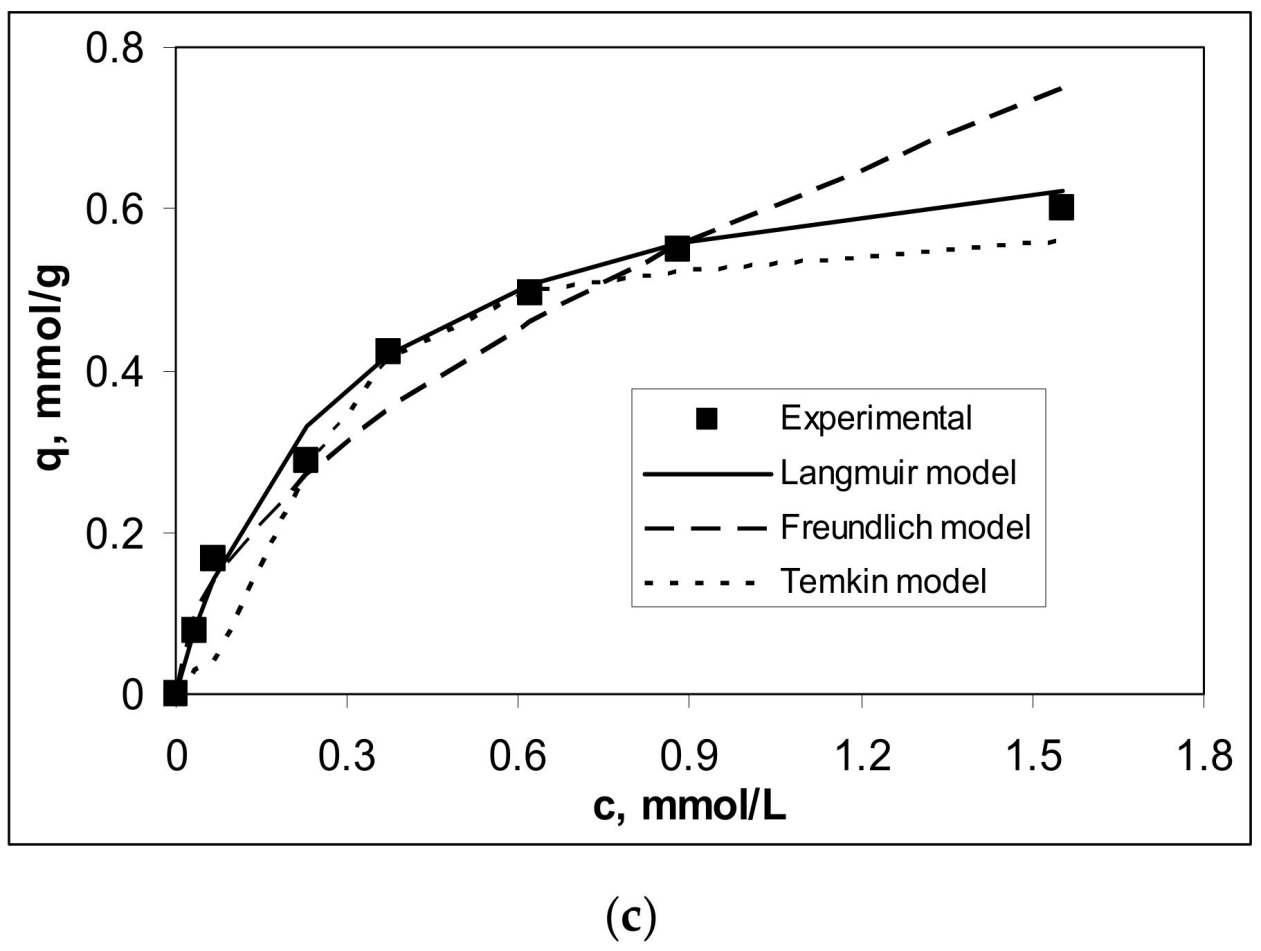
Experimental and theoretical isotherms obtained for the biosorption of (**a**) Cu(II), (**b**) Co(II) and (**c**) Zn(II) ions on Alg-Fe_3_O_4_-MPs. (Experimental conditions: pH = 5.4; adsorbent dosage = 2.0 g/L; contact time = 24 h; temperature = 23 °C).

The quantitative analysis of the equilibrium data of Cu(II), Co(II) and Zn(II) ions’ biosorption on Alg-Fe_3_O_4_-MPs was done using Langmuir, Freundlich and Temkin isotherm models. Figure 5 illustrates the experimental and calculated isotherms, based on the equations of the above isotherm models (Equations (6)–(8)), for each metal ion separately, while the values of the equilibrium parameters are summarized in Table 1.

The Langmuir model best describes the equilibrium data for the biosorption of Cu(II), Co(II) and Zn(II) ions on Alg-Fe_3_O_4_-MPs, compared to Freundlich and Temkin models (Figure 5). This indicates that the retention of metal ions takes place predominantly on the outer surface of the biosorbent, until monolayer coverage is formed. The maximum biosorption capacities (q_max_, mmol/g) (Table 1) are close to the experimental values obtained at the highest initial concentrations of metal ions (0.73 mmol/g for Cu(II), 0.38 mmol/g for Co(II) and 0.65 mmol/g for Zn(II)), which explains the tendency of flattening of experimental isotherms in the high concentration range (see Figure 5). 

In addition, these results support the observations made previously, namely that Alg-Fe_3_O_4_-MPs is an effective biosorbent only up to a certain value of the concentration of metal ions. Moreover, the Langmuir constants (K_L_, L/g), although having the same order of magnitude for all studied metal ions (Table 1), increase in the order: Cu(II) < Zn(II) < Co(II). The detailed analysis of these values shows that: (i) the same types of interactions are involved in the biosorption of Cu(II), Co(II) and Zn(II) ions on Alg-Fe_3_O_4_-MPs (these are probably ion-exchange taking into account the adsorption energy values obtained from Temkin model (Table 1)), and (ii) these interactions are stronger in the case of Cu(II) ions, followed by Zn(II) ions and then Co(II) ions (in agreement with the values of 1/n constant from Freundlich model (Table 1)).

The comparison of biosorption capacities of various biosorbents for Cu(II), Co(II) and Zn(II) ions biosorption, under similar experimental conditions, are presented in Table 2. It can be seen that Alg-Fe_3_O_4_-MPs has good biosorption performance compared to other biosorbents, which highlights its applicability in the decontamination processes of industrial effluents.

#### 3.2.3. Adsorption Thermodynamics

The dependencies illustrated in Figure 3d show that increasing the temperature from 7 to 40 °C increases the amount of metal ions retained on Alg-Fe_3_O_4_-MPs biosorbent in all cases. Even if this increase of the biosorption capacity with the increase of temperature is not a significant one, it is important for the thermodynamic description of the studied biosorption processes. Thus, variation of the Gibbs free energy (ΔG^0^) was evaluated using the Langmuir constant (K_L_, g/L), obtained from the experimental isotherm for each metal ion (Cu(II), Co(II) and Zn(II)) at each value of temperature (7, 23 and 40 °C), while the variation of enthalpy (ΔH^0^) and entropy (ΔS^0^) were obtained from the slope and intercept of the linear plots ln K_L_ vs. 1/T (data not shown). The values of the thermodynamic parameters calculated for the biosorption of each metal ion are summarized in Table 3.

The negative values of ΔG^0^ and the positive values of ΔH^0^ (Table 3) indicate that the retention of all metal ions (Cu(II), Co(II) and Zn(II)) on Alg-Fe_3_O_4_-MPs are spontaneous and endothermic. Therefore, the increase in temperature has a positive effect on the efficiency of these biosorption processes, which has been demonstrated experimentally (see Figure 3d). The relatively low values of ΔH^0^ (below 15 kJ/mol) [11] show that electrostatic interactions are predominant in the biosorption mechanism. Under these conditions, the selection of optimal experimental conditions (which ensures the speciation form of the metal ions and the dissociation state of the functional groups of the adsorbent) has a decisive role in the efficient realization of the biosorption processes. The positive values of ΔS^0^ indicate that all metal ions have a good affinity for Alg-Fe_3_O_4_-MPs, which results in increased randomness at the solid/liquid solution interface during the biosorption processes [39].

#### 3.2.4. Kinetics Modelling

**Figure 6:**
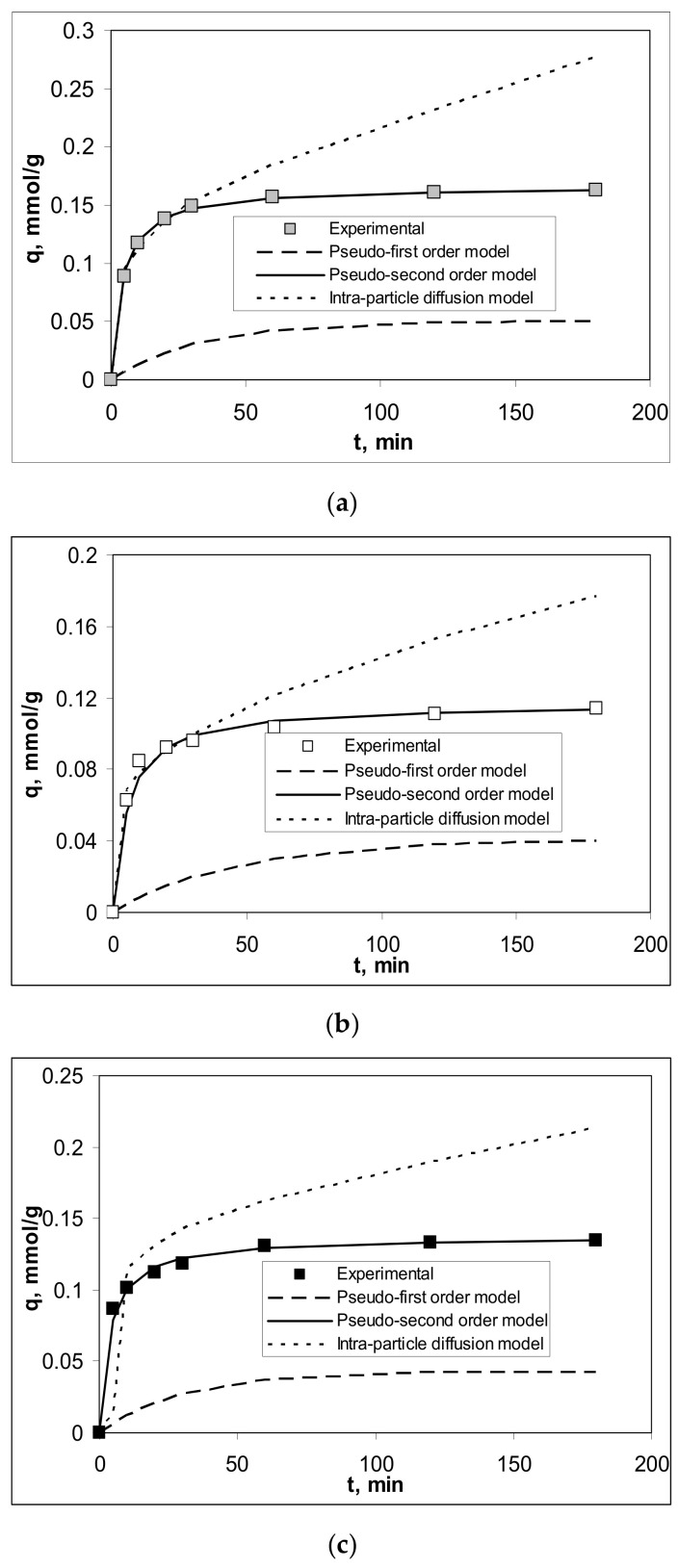
Experimental and theoretical kinetic curves obtained for the biosorption of (**a**) Cu(II), (**b**) Co(II) and (**c**) Zn(II) ions on Alg-Fe_3_O_4_-MPs. (Experimental conditions: pH = 5.4; adsorbent dosage = 2.0 g/L; initial metal ions concentration = 0.80 mmol/L; temperature = 23 °C).

The modelling of the kinetic behaviour of Cu(II), Co(II) and Zn(II) ions’ biosorption on Alg-Fe_3_O_4_-MPs was done using the pseudo-first order, pseudo-second order and intra-particle diffusion models. The most appropriate kinetic model to describe the experimental data was selected based on the values of the regression coefficients (R^2^), calculated for the statistical analysis. Figure 6 illustrates the experimental and calculated kinetic curves (based on the equations of these three kinetic models) for the biosorption of the studied metal ions on Alg-Fe_3_O_4_-MPs, while the values of the kinetic parameters are presented in Table 4.

The regression coefficients (R^2^) (Table 4) indicate that the experimental data are best described by the pseudo-second order equation for all studied metal ions, as is easily observed in Figure 6. Furthermore, the calculated biosorption capacities of this model (q_e_^calc^, mmol/g) are in good agreement with the experimental values (q_e_^exp^, mmol/g) (Table 4). These results illustrate that the biosorption of Cu(II), Co(II) and Zn(II) ions on Alg-Fe_3_O_4_-MPs is consistent with the pseudo-second order kinetic model. Therefore, these biosorption processes mainly take place through chemical interactions (most likely of the ion exchange type), and the retention of metal ions requires the existence of two binding sites [25]. This hypothesis is also supported by the values of the rate constants (Table 4). 

The values of the rate constants of the pseudo-second order model (k_2_, mmol/g min) increase in the order Cu(II) < Co(II) < Zn(II) (Table 4), which indicate that Zn(II) ions are more easily retained on the surface of the adsorbent compared to Cu(II) and Co(II) ions. This variation can be explained by considering the electronegativity values of metal ions [34]. Thus, Zn(II) ions, which have the lowest electronegativity (1.65), can participate more easily in ion exchange interactions than Co(II) (1.88) or Cu(II) (1.90) [34].

Modelling using the intra-particle diffusion model shows that this model describes the experimental data in the initial stage of the biosorption processes well (Figure 6). This suggests that elementary intra-particle diffusion processes contribute to the retention of Cu(II), Co(II) and Zn(II) ions on Alg-Fe_3_O_4_-MPs. However, the linear representations of q_t_ vs. t^1/2^ do not pass through the origin (data not shown) for any of the studied metal ions, which means that intra-particle diffusion is not the rate-limiting step in these adsorption processes [25].

These elementary intra-particle diffusion processes ensure that only metal ions can reach the vicinity of the functional groups of the biosorbent, thus facilitating the possibility of their participation in chemical interactions. In this way, it can be explained why the rate constants of the first region (k_diff_^1^, mmol/g min^1/2^) corresponding to the film diffusion are much larger compared to the rate constants of the second region (k_diff_^2^, mmol/g min^1/2^), which describes the diffusion of metal ions into the pores of Alg-Fe_3_O_4_-MPs (Table 4).

### 3.3. Biosorption Mechanism

**Figure 7:**
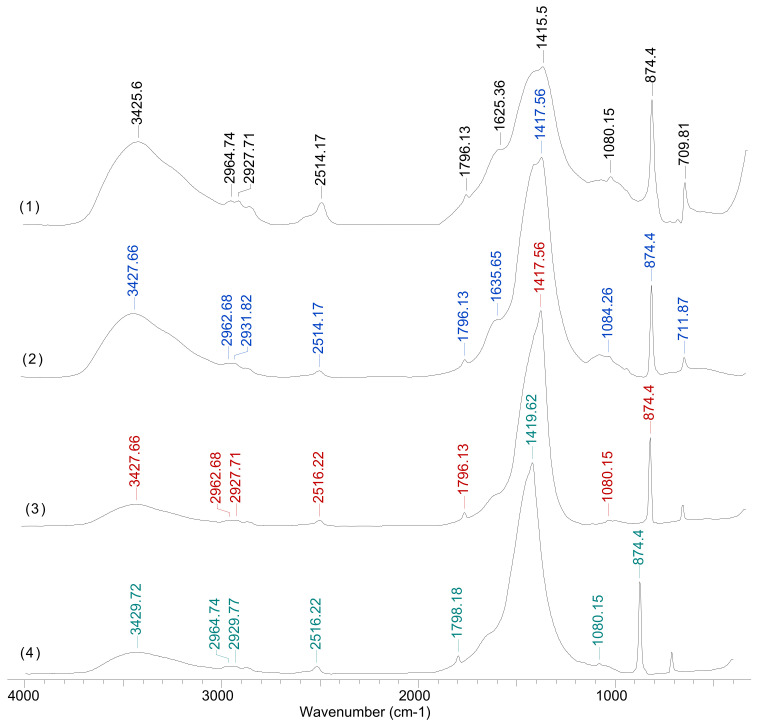
FTIR spectra of Alg-Fe_3_O_4_-MPs before (1) and after biosorption of Cu(II) (2), Co(II) (3) and Zn(II) (4) ions from aqueous media.

The analysis of the biosorption mechanism is important because it can provide a theoretical basis for the use of Alg-Fe_3_O_4_-MPs in metal ions removal processes, and can highlight its practical applicability. Thermodynamic and kinetic modelling (presented in the previous sections) shows that: (i) metal ions retention takes place in a monolayer until the adsorbent surface is completely covered (according to the Langmuir isotherm model), after which the adsorption processes reach equilibrium; and (ii) the interactions between the metal ions and the functional groups of the adsorbent are predominantly of the ion exchange type (see Table 4), the binding of each metal ion requiring two binding sites that have a favourable geometric position (according to the pseudo-second order kinetic model). To support these observations, the FTIR spectra of Alg-Fe_3_O_4_-MPs were recorded before and after retention of each metal ion (Figure 7). 

After adsorption of Cu(II), Co(II) and Zn(II) ions no new additional absorption bands appear in the FTIR spectra (Figure 7). Therefore, the binding of metal ions to the functional groups of Alg-Fe_3_O_4_-MPs does not involve new covalent bonds, implying a mechanism of superficial complexation. The characteristic absorption bands are shifted by 4–10 cm^−1^ (see Table 5) after the retention of metal ions, which suggests that their binding takes place through predominantly electrostatic interactions. 

These observations have two important practical consequences, namely: (1) the selectivity of the biosorption process using Alg-Fe_3_O_4_-MPs will be determined mainly by the structural characteristics of metal ions from aqueous solution (ionic radius, electronegativity, etc.); and (2) the desorption of metal ions takes place by treating the biosorbent load with metal ions with a mineral acid (HCl, HNO_3_, etc.) or complexing agent such as EDTA.

### 3.4. Desorption and Reusability

**Figure 8:**
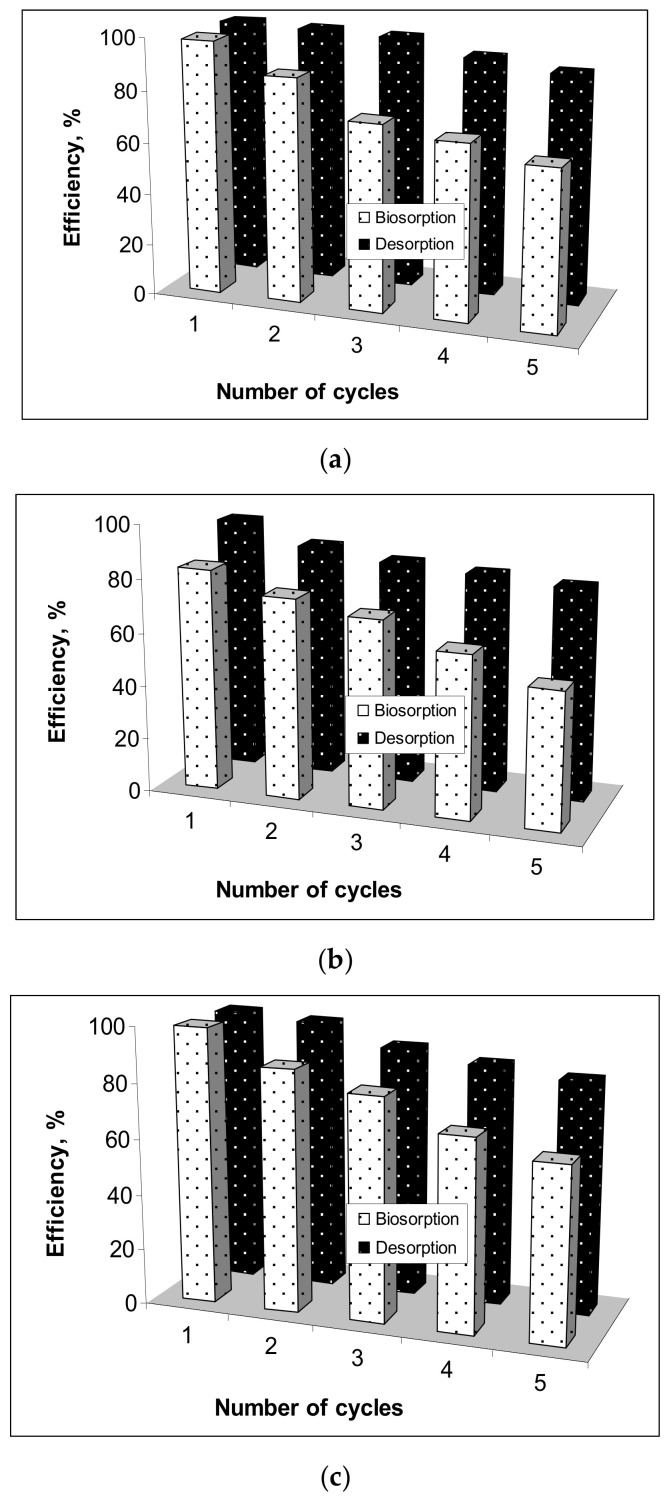
Efficiency of biosorption/desorption processes depending on number of cycles for (**a**) Cu(II), (**b**) Co(II) and (**c**) Zn(II).

According to the previous observations, desorption of metal ions (Cu(II), Co(II) and Zn(II) ions) from loaded Alg-Fe_3_O_4_-MPs was done using a 0.1 mol/L HNO_3_ solution. The reusability of Alg-Fe_3_O_4_-MPs was tested in five consecutive biosorption/desorption cycles, using the same amount of biosorbent for each metal ion (Figure 8).

As can be seen from Figure 8, the increase in the number of cycles determined a slight decrease of desorption efficiency (14.47%) and a more accentuated decrease of biosorption efficiency (34.96%) for all studied metal ions. The more pronounced decrease in the efficiency of biosorption processes with increasing number of cycles is mainly due to the fact that after treatment with HNO_3_ (for desorption) some of the functional groups become unavailable for the retention of metal ions, either due to the formation of H-bonds or due to their destruction. However, at the end of the five cycles the desorption percentages of metal ions (Cu(II), Co(II) and Zn(II)) were higher than 75% indicating that Alg-Fe_3_O_4_-NPs may be considered a reusable material, which is effective in removing metal ions from aqueous media.

## 4. Conclusions

In this study, Alg-Fe_3_O_4_-MPs biosorbent was obtained by in situ functionalization of iron oxide particles with alginate extracted from marine algae biomass. In this way, a complete removal of the alginate from the solution after extraction was ensured and a new material with biosorptive properties could be obtained. To confirm biosorptive potential, Alg-Fe_3_O_4_-MPs was tested for the removal of Cu(II), Co(II) and Zn(II) ions from aqueous media. Under optimal experimental conditions established with batch systems (pH of 5.4; adsorbent dosage of 2 g/L; contact time of minimum 60 min and room temperature (23 ± 1 °C)), the efficiency of metal ions removal was higher of 90% when the initial metal ions concentration was less than 0.8 mmol/L. In addition, biosorption processes have been shown to be spontaneous (ΔG^0^ < 0), endothermic (ΔH^0^ > 0), and best described by the Langmuir isotherm and the pseudo-second order kinetic models. The respective maximum biosorption capacities (q_max_, mg/g) follow the order Cu(II) > Zn(II) > Co(II), and are comparable to the values reported in the literature for retention of these ions on other biosorbents. Analysis of the kinetic parameters shows that the retention of Cu(II), Co(II) and Zn(II) ions on the Alg-Fe_3_O_4_-MPs most likely takes place predominantly through an ion exchange mechanism; this hypothesis is also supported by the results of desorption studies. The very good biosorptive and regenerative performance of Alg-Fe_3_O_4_-MPs highlights the potential of this material for the treatment of wastewater containing metal ions.

## Figures and Tables

**Table 1:** Isotherm parameters for Cu(II), Co(II) and Ni(II) ions biosorption on Alg-Fe_3_O_4_-MPs.

Isotherm Parameter		Cu(II)	Co(II)	Zn(II)
Langmuir model	R^2^	0.9819	0.9877	0.9851
	q_max_, mmol/g	0.7262	0.3842	0.6528
	K_L_, L/mmol	2.8151	5.3866	3.6127
Freundlich model	R^2^	0.9302	0.9089	0.9481
	n	3.73	1.88	2.34
	K_F,_ L^1/n^/g·mmol^1/(n−1)^	0.7369	0.2835	0.5927
Temkin model	R^2^	0.9599	0.9695	0.9002
	A_T_, L/g	0.2852	0.3761	0.3847
	B, kJ/mol	20.82	17.46	19.14

**Table 2:** Comparison of the maximum biosorption capacity (q_max_, mmol/g) of Alg-Fe_3_O_4_-MPs for Cu(II), Co(II) and Zn(II) ions with similar algae-based materials.

Adsorbent	Cu(II)	Co(II)	Zn(II)	References
*Ulva fasciata* sp.	0.42	-	0.21	[35]
*Callithamnion corymbosum* sp.	0.38	0.17	0.29	[17]
Calcium-alginate	1.02	0.32	0.56	[17]
Alginate/polyethylenimine	1.14	-	0.70	[36]
Titania-coated silica/alginate	0.32	-	0.27	[37]
Fe_2_O_3_@Microalgae	0.61	-	-	[38]
Commercial Fe_2_O_3_	0.16	-	-	[38]
Alg-Fe_3_O_4_-MPs	0.73	0.38	0.65	This study

**Table 3:** Thermodynamic parameters obtained at the biosorption of Cu(II), Co(II) and Zn(II) ions on Alg-Fe_3_O_4_-MPs.

Metal ion	ΔG^0^, kJ/mol	ΔH^0^, kJ/mol	ΔS^0^, J/mol K
Cu(II)	−42.03	15.57	192.43
Co(II)	−42.39	14.03	189.05
Zn(II)	−53.46	14.89	228.85

**Table 4:** Kinetic parameters for Cu(II), Co(II) and Ni(II) ions biosorption on Alg-Fe_3_O_4_-MPs.

Kinetics Parameter			Cu(II)	Co(II)	Zn(II)
q_e,exp_, mmol/g			0.1624	0.1141	0.1344
Pseudo-first order		R^2^	0.9132	0.9639	0.9539
		q_e,calc_ mmol/g	0.0509	0.0408	0.0430
		k_1_, 1/min	0.0130	0.0098	0.0143
Pseudo-second order		R^2^	0.9999	0.9995	0.9999
		q_e,calc_ mmol/g	0.1661	0.1167	0.1372
		k_2_, g/mmol min	1.5223	1.5843	1.9794
Intra-particle diffusion model	Zone 1	R^2^	0.9516	0.8485	0.9573
		c, mmol/L	0.0540	0.0468	0.0676
		k_diff_^1^, mmol/g min^1/2^	0.0181	0.0097	0.0097
	Zone 2	R^2^	0.9921	0.9663	0.9856
		c, mmol/L	0.1496	0.0892	0.1266
		k_diff_^2^, mmol/g min^1/2^	0.0010	0.0019	0.0006

**Table 5:** Variation of the maximum wave numbers of the characteristic peaks in the FTIR spectra of Alg-Fe_3_O_4_-MPs before and after biosorption of metal ions.

Before Biosorption, Alg-Fe_3_O_4_-MPs	After Biosorption		
	Cu(II)	Co(II)	Zn(II)
3426.6 cm^−1^	3427.66 cm^−1^	3427.66 cm^−1^	3429.72 cm^−1^
2964.74 cm^−1^	2962.68 cm^−1^	2962.68 cm^−1^	2964.74 cm^−1^
2927.71 cm^−1^	2931.82 cm^−1^	2927.71 cm^−1^	2929.77 cm^−1^
2514.17 cm^−1^	2514.17 cm^−1^	2516.22 cm^−1^	2516.22 cm^−1^
1796.13 cm^−1^	1796.13 cm^−1^	1796.13 cm^−1^	1796.13 cm^−1^
1415.15 cm^−1^	1417.56 cm^−1^	1417.56 cm^−1^	1419.62 cm^−1^
1080.15 cm^−1^	1084.26 cm^−1^	1080.15 cm^−1^	1080.15 cm^−1^
874.40 cm^−1^	874.40 cm^−1^	874.40 cm^−1^	874.40 cm^−1^

## Data Availability

Not applicable.
